# Pharmacologic antagonism of CB1 receptors improves electrophysiological alterations in Purkinje cells exposed to 3-AP

**DOI:** 10.1186/s12868-023-00786-4

**Published:** 2023-03-03

**Authors:** Hoda Ranjbar, Monavareh Soti, Kristi A. Kohlmeier, Mahyar Janahmadi, Mohammad Shabani

**Affiliations:** 1 Neuroscience Research Center, Neuropharmacology Institute, Kerman University of Medical Sciences, Kerman, 76198-13159 Iran; 2 Department of Drug Design and Pharmacology, Faculty of Health Sciences, University of Copenhagen, Copenhagen, Denmark; 3 Department of Physiology, School of Medicine, Shahid Beheshti University of Medical Sciences, Tehran, Iran

**Keywords:** Ataxia, Cerebellum, Purkinje cell, Cannabinoid, CB1R

## Abstract

**Introduction:**

Although ataxia is associated with cerebellar dysfunction, little is known about the effects of 3-AP exposure on Purkinje cell electrophysiological properties. Here, we evaluated these parameters in cerebellar vermis brain slices.

**Methods:**

Purkinje cells were exposed to artificial cerebrospinal fluid (aCSF) (control) or to 1 mM 3-acetylpyridine (3-AP) in the recording chamber. The effects of a cannabinoid agonist (WIN; 7.5 nmol) and a cannabinoid antagonist (AM; 20 nmol) were evaluated under both conditions.

**Results:**

Exposure to 3-AP induced dramatic changes in cellular excitability that likely would affect Purkinje cell output. In whole-cell current clamp recordings, 3-AP-exposed Purkinje cells demonstrated a significantly higher frequency of action potentials, a larger afterhyperpolarization (AHP), and a larger rebound of action potentials. In addition, 3-AP caused a significant decrease in the interspike interval (ISI), half-width, and first spike latency. Remarkably, the action potential frequency, AHP amplitude, rebound, ISI, action potential halfwidth, and first spike latency were no longer different from controls in 3-AP cells treated with AM. Sag percentage, on the other hand, showed no significant difference under any treatment condition, indicating that cannabinoids' actions on 3-AP-mediated Purkinje cell changes may not include effects on neuronal excitability through changes of Ih.

**Conclusions:**

These data show that cannabinoid antagonists reduce the excitability of Purkinje cells following exposure to 3-AP and suggest their potential as therapeutics in cerebellar dysfunctions.

## Highlights

- Purkinje cell excitability dramatically increased after exposure to 3-AP.
- 3-AP caused a significant decrease in ISI, half-width, and first spike latency.
- CB1R antagonist attenuated the cellular effects of 3-AP.
- AM251 reduced the excitability of Purkinje cells exposed to 3-AP.

## Introduction

Cerebellar ataxia is considered a diverse group of neurological disorders characterized by loss of balance and motor coordination caused by abnormal neuronal functioning in the cerebellum [1]. In addition to acquired causes (such as brain trauma, infections, ischemia, alcohol misuse, or other factors) [2], ataxias can also be inherited [3]. The cerebellar Purkinje cells are part of a complex circuit that integrates information from many sources and provides the sole output from the cerebellar cortex. Like other neuronal types, the activity of Purkinje cells is regulated by synaptic inputs, which include excitatory inputs from parallel and climbing fibers and inhibitory inputs from basket and stellate cells [4].

Animal models of ataxia include genetic mutations, but 3-acetyl pyridine (3-AP) administration to rats can also produce symptoms of ataxia, such as severe motor coordination and locomotor activity impairment [5, 6]. The inferior olive is the only target of climbing fiber afferents to the cerebellum, and 3-AP affects the inferior olive through impairing electron transport [7]. As a result, because climbing fibers modulate Purkinje cell responses, impaired climbing fibers alter Purkinje cell activity [8, 9]. Since Purkinje cells are the cerebellar cortex's only output neuron, under conditions of ataxia that lead to Purkinje cell degeneration, it is important to understand the impact of the impaired climbing fibers on intrinsic activity of Purkinje cells prior to the death of these cells [7]. In addition to previously recognized effects on the inferior olive, the 3-AP has direct effects on Purkinje cells, which change electrophysiological properties and ultimately lead to Purkinje cell death [10].

The endocannabinoid system is comprised of receptors, endogenous agonists, and related biochemical mechanisms that synthesize and terminate the actions of endogenous agonists. CB1 and CB2 receptors were named after the receptors activated by cannabinoids in the order of discovery [11]. CB1 receptors (CB1Rs) are mainly expressed in the CNS, and their density is particularly high in the cerebellum, suggesting that they play a significant role in cerebellum function, which is consistent with findings that Purkinje cell synaptic transmission is modulated by CB1R activation at excitatory and inhibitory synapses [12].

In spinocerebellar ataxias (SCAs), the endocannabinoid system becomes dysregulated in the cerebellum and in other parts of the central nervous system (e.g., brainstem, basal ganglia), which contributes to the progression of pathogenic events. Based on the results of studies conducted in rodent models and postmortem tissue analysis, changes in CB1Rs appear to be responsible for acutely modulating motor incoordination in cerebellar ataxias [13, 14]. Additional studies in post-mortem tissues from patients suffering from SCA have also demonstrated elevated levels of CB1Rs in Purkinje neurons, with a similar profile found for endocannabinoid hydrolyzing enzymes [13, 15]. When taken together, these studies and others suggest that activating CB1Rs and/or inhibiting these enzymes could serve to develop cannabinoid-based neuroprotective therapies. Therefore, the endocannabinoid system is a current focus of studies examining treatment approaches for ataxia [15–18].

Changes in endocannabinoid transmission could affect the firing of Purkinje cells prior to their death in ataxia. However, as studies of Purkinje cell electrical activity associated with ataxia have been conducted with extracellular recordings, information is lacking regarding the effect(s) of ataxia on the cellular electrophysiology of cerebellar neurons prior to death. Therefore, since the 3-AP model is useful for studying mechanisms underlying changes in Purkinje cells activity associated with ataxia, the role played by endocannabinoid transmission in dysfunctions involving changes in Purkinje cell properties can be studied in this model. Further, the effects of administration of agonist/antagonist of cannabinoid receptors have not been shown in this model. Accordingly, this study characterized changes in the electrophysiological activity of cerebellar Purkinje cell exposed to 3-AP prior to their inevitable death. In addition, the effects of agonist/antagonists of cannabinoid receptors on changes in Purkinje cell function exposed to 3-AP were examined to evaluate whether cannabinoids could offer a potential therapy for cerebellar dysfunction.

## Method and material

### Animals

Experiments were performed on tissue obtained from 36 male Wistar rats (weighing 30–60 g) aged 4–6 weeks at the start of the protocol, obtained from the Kerman University of Medical Sciences, and housed under controlled photoperiod (lights on: 07:00–19:00 h), at 22 ± 1 °C with food and water available. All protocols and procedures using animals were approved by the Kerman University of Medical Sciences Ethic Committee (IR.KMU.REC.1399.254). Male rats (n = 6 cells/group; one cell from each rat) were divided randomly into six groups: control (Co), cannabinoid agonist (WIN; 7.5 nmol) [19], cannabinoid antagonist (AM; 20 nmol) [19], 3 acetyl pyridine (3-AP; 1 mM) [20], 3-AP + WIN, and 3-AP + AM.

### Preparation of cerebellar slices

Rats were euthanized by cervical dislocation, and the posterior skull was removed allowing extraction of the cerebellum in the coronal plane. The brain was sliced into sections of a 250 μm thickness using a Vibroslicer (Campden Instrument, NVSLM1, Sarasota, FL) in artificial cerebrospinal fluid (aCSF) containing (in mM) 124 NaCl, 25 NaHCO3, 10 D-glucose, 4.4 KCl, 2 MgCl2, 1.25 NaH2PO4, and 2 CaCl2, which was bubbled with 95% O2–5% CO2 (pH 7.4 ± 0.05 and osmolarity was adjusted to 300 ± 10 mOsm). The slices were immediately incubated in aCSF at 35 ± 2 °C for 30 min before being recorded within 6 h at room temperature (22 °C).

### Whole cell patch clamp

A Multiclamp 700B amplifier from Axon Instruments was used to conduct whole-cell patch clamp recordings from Purkinje cells in current clamp and voltage clamp modes. Purkinje cells were identified using the established criteria listed below. Signals were digitized using a 1440 A/D converter (Axon Instruments). Electrophysiological data were sampled at 10 kHz and filtered at 20 kHz [21]. Patch pipettes had a resistance of 5–9 MΩ when filled with an internal solution comprising (in mM) 140 potassium gluconate, 5 KCl, 10 HEPES, 2 MgCl2, 0.2 EGTA, 2 Na2ATP, and 0.4 Na2GTP. The internal solution's pH and osmolarity were adjusted to 7.3 (by KOH) and 290 mOsm, respectively. The perfusion rate of the recorded slides was 1.9–2 ml/min. Following the formation of a giga seal on the cell membrane, a brief suction was used to rupture the membrane, allowing the cell to be clamped to a holding voltage of -60 mV. The test seal function was continuously used throughout the recording to make sure the seal was stable. The number of action potentials generated after negative current injection when the cell rebounded to the resting membrane potential, and the spike latency of the first action potential were measured. In spontaneously firing neurons, action potential parameters including action potential (AP) half-width, AP frequency, AP amplitude, AHP amplitude, voltage threshold (the membrane potential from the base and start of the action potential), interspike interval (ISI), and coefficient of variation (CV) (regularity of action potential and dispersion of a probability/frequency) were measured [22].

The sag voltage in response to hyperpolarizing current pulses (amplitude of -100 pA to -500 pA) was calculated. The peak voltage deviation was divided by the amplitude of the steady-state voltage deviation using the following formula:

Sag voltage = Vpeak − Vsteady state.

Furthermore, the sag was calculated as [(peak response—steady state response) / peak response] 100. To examine the effect of a CB1R agonist/antagonist on Ih in Purkinje cells, I-V activation curves were obtained in voltage clamp mode using 520 ms of hyperpolarizing steps (50 to 140 mV in increments of 10 mV).

### Drug application

Drugs were applied in aCSF from a separate reservoir (also gassed with carbogen) to the recording chamber. 3-AP (Sigma, USA) was dissolved in deionized water as a × 100 stock solution containing 0.4% ascorbic acid. Purkinje cells were exposed to aCSF (control) or 1 mM 3-AP (for at least 20 min). WIN (7.5 nmol) and AM (20 nmol) were added to the bath [23].

### Statistical analysis

Data are presented as mean ± SEM using Graph Pad Prism 9 (Graph Pad Software, USA). Normally distributed data were compared by a two-way ANOVA, and the Tukey post hoc analysis was used for multiple comparisons between multiple groups. Differences were considered statistically significant when P < 0.05.

## Result

### The effect of CB1R agonist/antagonist on the electrophysiological activities of Purkinje cells following exposure of 3-AP in cerebellum slices

**Fig. 1:**
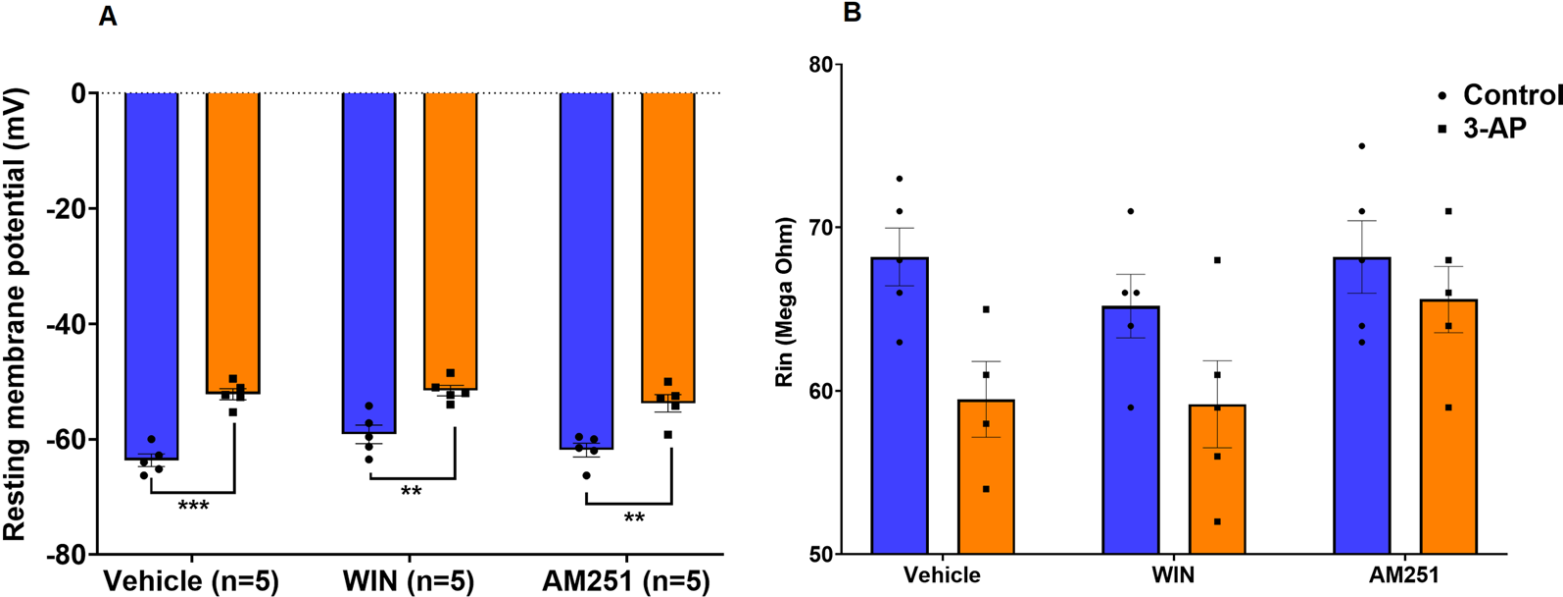
Changes in electrophysiological properties of Purkinje cells in vermis slices treated with CB1R agonist/antagonist and 3-AP. **A** 3-AP alone and in combination with CB1R agonist/antagonist resulted in a lower RMP compared to control, WIN, and AM respectively. **B** No significant differences were observed in Rin. n = 6 cells/group; one cell from each rat. The data are expressed as mean ± SEM. **(P < 0.01) and ***(P < 0.001) represent a significant difference with the counterpart group

In CB1R agonist/antagonist and 3-AP-exposed slices, a total of 30 Purkinje cells were recorded. The passive and active properties of 6 cells were examined for each group. Our results indicated that cells in the 3-AP (P < 0.001), 3-AP + WIN (P < 0.01), and 3-AP + AM (P < 0.01) treated groups exhibited a significantly lower resting membrane potential (RMP) (F (2, 24) = 1.446, P = 0.2554) compared to cells in the Co, WIN, and AM groups, respectively (Fig. 1A). We measured R_in_ as an index of how the cells would passively respond to current inputs. There were no statistical differences in R_in_ (F (2, 23) = 0.9682, P = 0.3947) between any of the Purkinje cell treatment groups, indicating that exposure to 3-AP whether in the presence or absence of cannabinoid agonists or antagonists, had no effect on R_in_ (Fig. 1B).

**Fig. 2:**
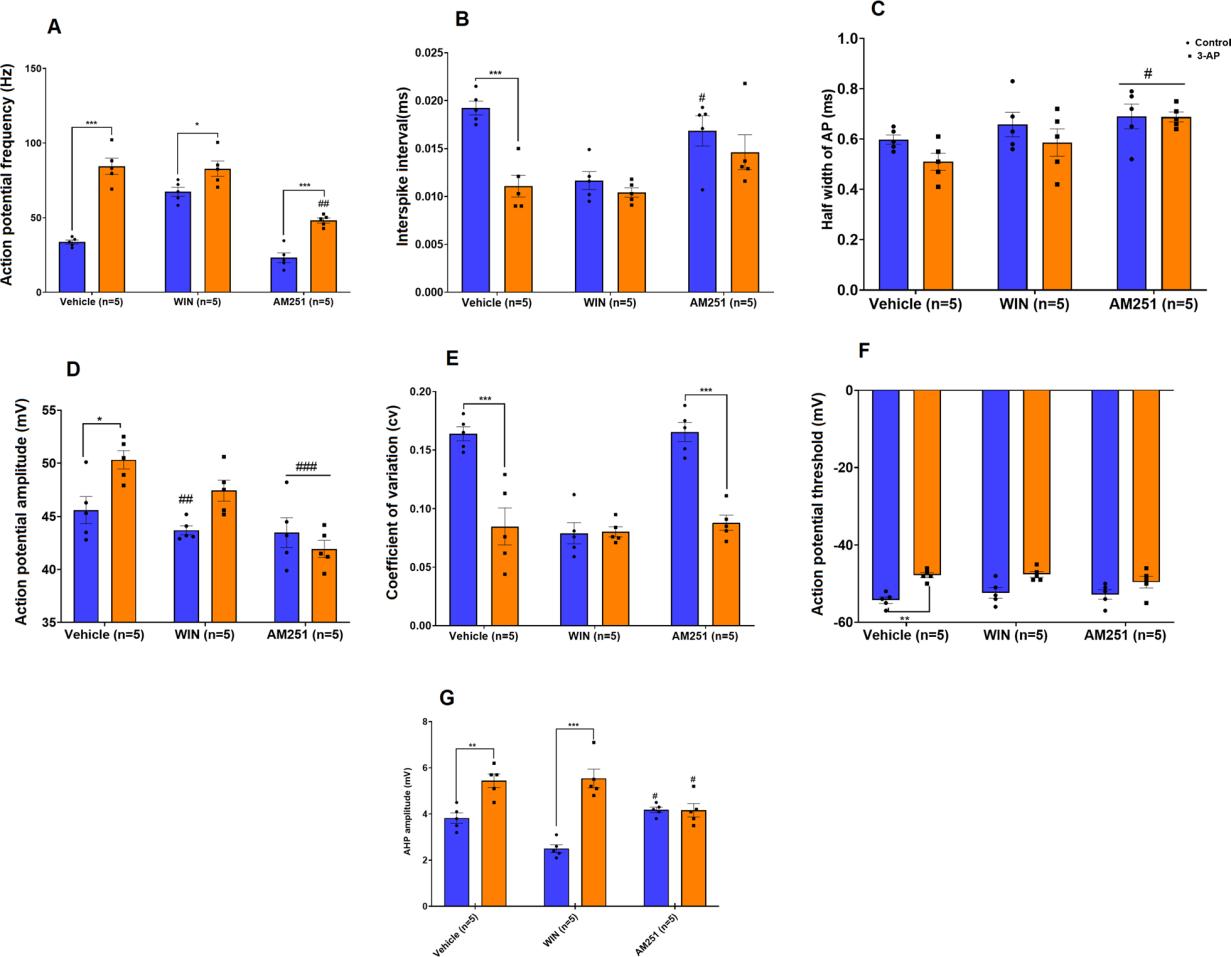
The effect of CB1R agonist/antagonist on the spontaneous firing properties of Purkinje cells in control and 3-AP treated slices. **A** Electrophysiological properties shown are action potential frequency, **B** interspike interval, **C** action potential half-width, **D** action potential amplitude, **E** coefficient variant, **F** action potential threshold, and **G** AHP amplitude. n = 6 cells/group; one cell from each rat. The data are expressed as mean ± SEM. *(P < 0.05), **(P < 0.01), and ***(P < 0.001) represent a significant difference with the counterpart group. #(P < 0.05), ## (P < 0.01), and ###(P < 0.001) represent a significant difference versus 3-AP

**Fig. 3:**
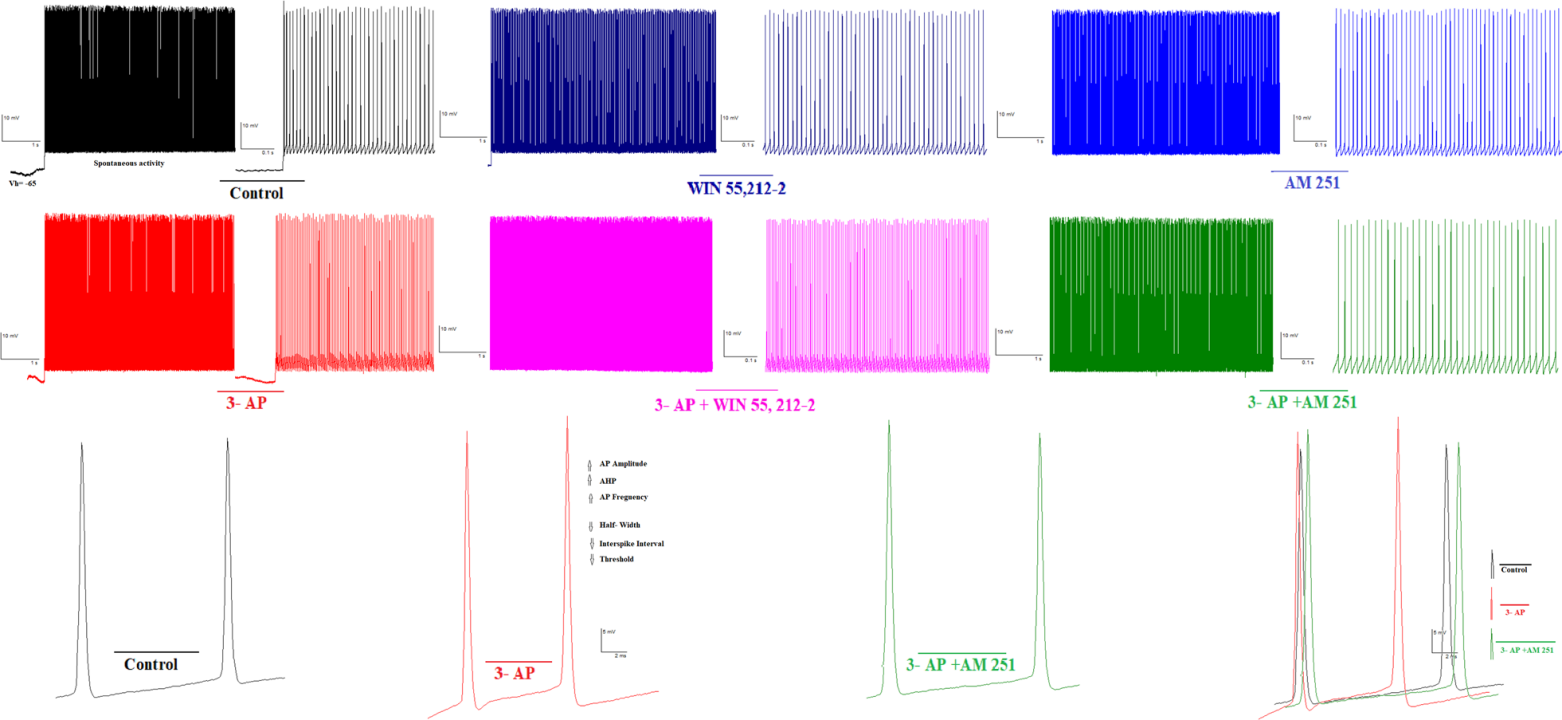
Representative traces of spontaneous firing from control and CB1R agonist/antagonist are depicted

Purkinje cells in 3-AP (P < 0.001), 3-AP + WIN (P < 0.05) and 3-AP + AM (P < 0.001) exposed slices demonstrated a higher spontaneous firing frequency (F (2, 24) = 12.41, P = 0.0002) when compared to that in the Co, WIN, and AM groups, respectively; whereas, Purkinje cells in the 3-AP + AM (P < 0.01) group demonstrated a significantly lower firing frequency when compared to that in 3-AP-treated cells (Fig. 2A). Besides firing rate, another important parameter of the Purkinje cell’s function is the interspike interval (ISI). We examined this parameter by measuring the spike intervals in Purkinje cells that were spiking spontaneously. This analysis indicated that cells exposed to 3-AP (P < 0.001) demonstrated a lower ISI (F (2, 24) = 4.817, P = 0.0174) compared to the Co and AM (P < 0.05) treated groups (Fig. 2B). We also found that Purkinje cells in the AM and 3-AP + AM (P < 0.05) groups had significantly broader AP half-widths (P < 0.05) compared to those seen in the 3-AP group (Fig. 2C). The amplitude of the action potential was significantly larger in Purkinje cells in the 3-AP group compared to cells in the Co group (P < 0.05). However, our data showed a lower amplitude of the action potential in cells from the WIN (P < 0.01), AM (P < 0.001), and 3-AP + AM (P < 0.001) groups compared to the amplitude in Purkinje cells of the 3-AP group (Fig. 2D). Purkinje cells in 3-AP (P < 0.001) exposed cell groups showed a lower CV (F (2, 24) = 12.67, P = 0.0002) compared to cells in the Co. In addition, 3-AP + AM (P < 0.001) cells showed lower CV compared to that seen in cells in the AM group (Fig. 2E). We examined the voltage threshold (F (2, 24) = 1.112, P = 0.3454) and kinetics of action potentials to determine if 3-AP affects these parameters. Purkinje cells from 3-AP-exposed (P < 0.01) cells demonstrated a significantly lower voltage threshold compared to cells in the Co group (Fig. 2F). The prominent AHP seen in Purkinje cells following an action potential reflects conductance activated during the spike. Amplitudes of the AHP (F (2, 24) = 16.49, P < 0.0001) in the Purkinje cells of the 3-AP (P < 0.01) and 3-AP + WIN groups (P < 0.001) were larger in amplitude compared to amplitudes seen in Co and WIN cells, respectively. Purkinje cells in the AM (P < 0.05) and 3-AP + AM (P < 0.05) groups displayed a significantly lower AHP compared to that seen in the 3-AP group (Fig. 2G). Furthermore, traces of spontaneous activity can be seen in Fig. 3, where two action potentials from each group have been selected and then superimposed. Compared to the Co and 3-AP + AM groups, the AP amplitude, AHP, and AP frequency were significantly higher, and the half width, and threshold were lower in the 3-AP groups (Fig. 3).

**Fig. 4:**
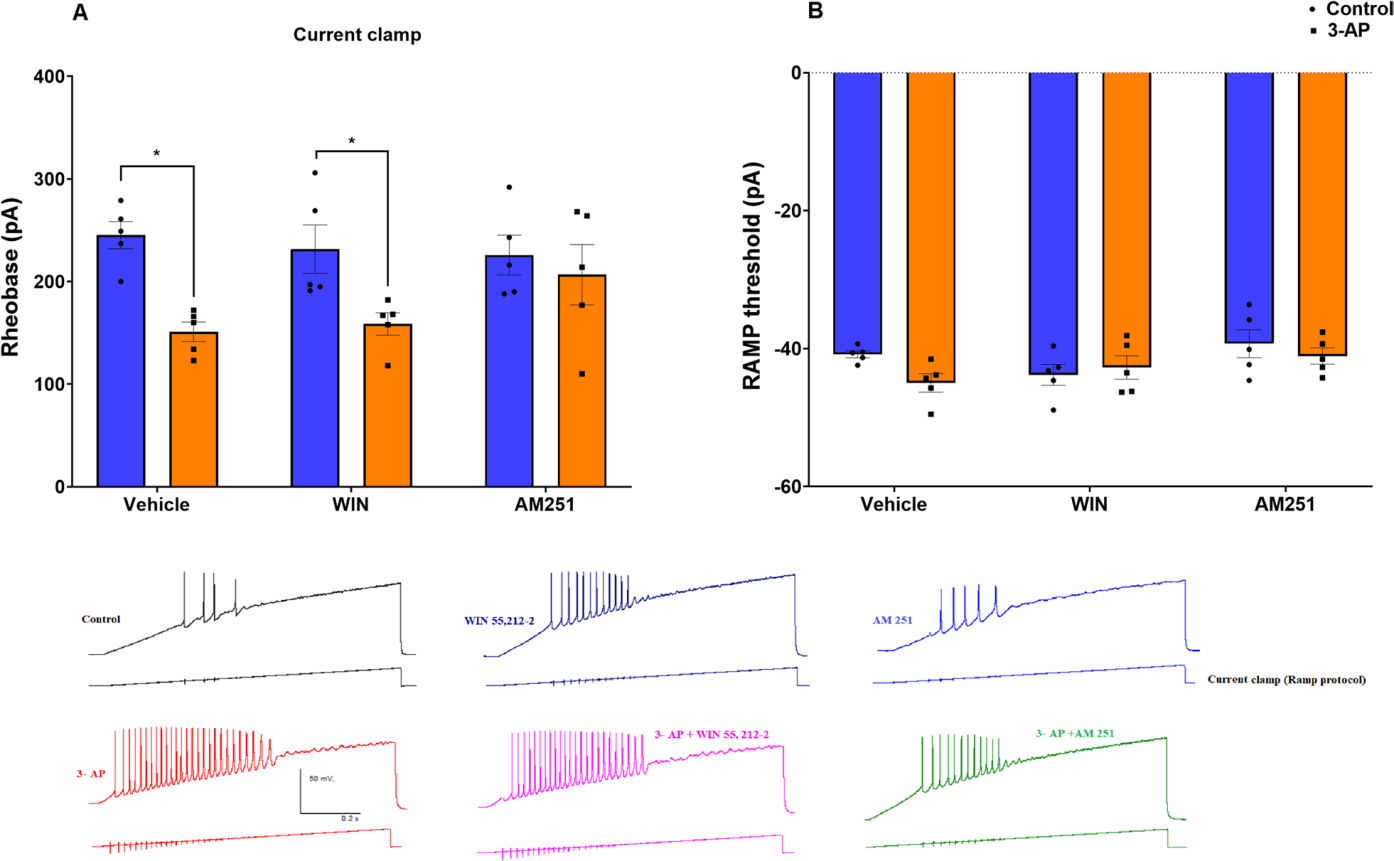
3-AP causes a reduction in rheobase in cerebellar Purkinje cells in current clamp recordings. **A** Rheobase (current clamp), **B** RAMP threshold. n = 6 cells/group; one cell from each rat. The data are expressed as mean ± SEM. *(P < 0.05) represent a significant difference with the counterpart group

To determine if 3-AP affects the excitability of cerebellar Purkinje cells, we first measured the rheobase. Rheobase is operationally defined as the minimal amount of electrical current required to trigger an action potential when current is injected into a cell. In current clamp mode, Purkinje cells exposed to 3-AP (P < 0.05) and 3-AP + WIN (P < 0.05) showed a significantly lower rheobase (F (2, 24) = 2.056, P = 0.1499), indicating a heightened excitability compared to cells in the Co and WIN groups, respectively (Fig. 4A). However, while the 3-AP and 3-AP + WIN groups required less current to stimulate an action potential, there was not a significant difference noted in the threshold voltage noted in the RAMP protocol, which indicates the current at which an action potential was triggered (F (2, 24) = 1.615, P = 0.2198) (Fig. 4B).

**Fig. 5:**
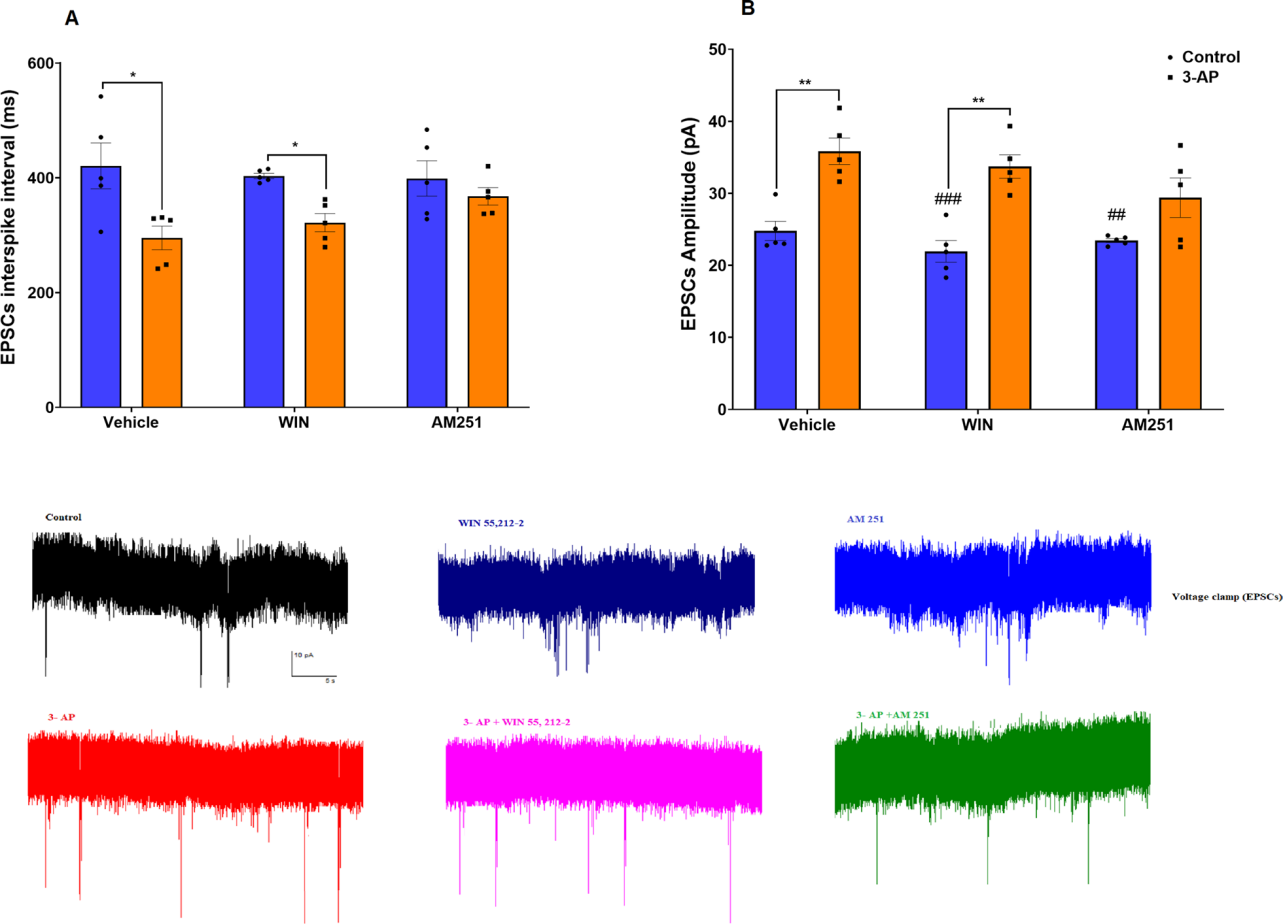
EPSPCs of Purkinje cells in slices exposed to CB1R agonist/antagonist in control and 3-AP treated slices. **A** EPSPCs interspike interval, **B** EPSCs amplitude is shown. n = 6 cells/group; one cell from each rat. The data are expressed as mean ± SEM. *(P < 0.05) and **(P < 0.01) represent significant differences with the same group with Vehicle (DMSO). #(P < 0.05) represents a significant difference versus 3-AP

We next examined the response of Purkinje cells to the effects of CB1R agonist/antagonist on spontaneous excitatory synaptic activity following exposure to 3-AP. Purkinje cells in the 3-AP (P < 0.05) and 3-AP + WIN (P < 0.05) groups showed smaller intervals of sEPSCs (F (2, 24) = 1.926, P = 0.1676) when compared to the sEPSCs intervals seen in the Co and WIN groups, respectively (Fig. 5A). Consistent with a heightened increase in excitability, the amplitude of sEPSCs (F (2, 24) = 1.717, P = 0.2009) in the Purkinje cells of the 3-AP group (P < 0.01) was increased. In addition, the combination of 3-AP and WIN (P < 0.01) amplified the action of 3-AP in enhancing sEPSP amplitudes. In Purkinje cells in the WIN (P < 0.001) and AM (P < 0.01) groups, there was a significantly lower amplitude of sEPSCs when compared to amplitudes seen in 3-AP cells. Administration of AM failed to significantly alter the effects of 3-AP on sEPSP amplitudes (Fig. 5B).

**Fig. 6:**
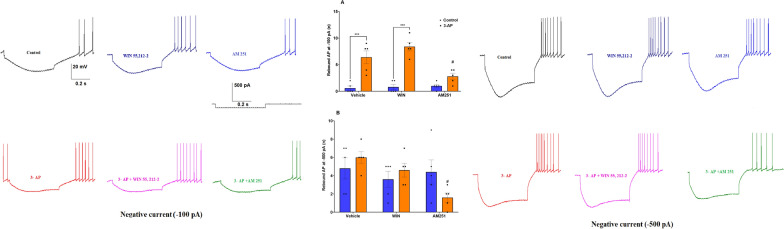
The effect of CB1R agonist/antagonist on the rebound of action potential at **A **-100pA and **B **-500 pA. n = 6 cells/group; one cell from each rat. The data are expressed as mean ± SEM. ***(P < 0.001) represents the significant difference with the same group with Vehicle (DMSO). #(P < 0.05) represents the significant difference versus 3-AP

**Fig. 7:**
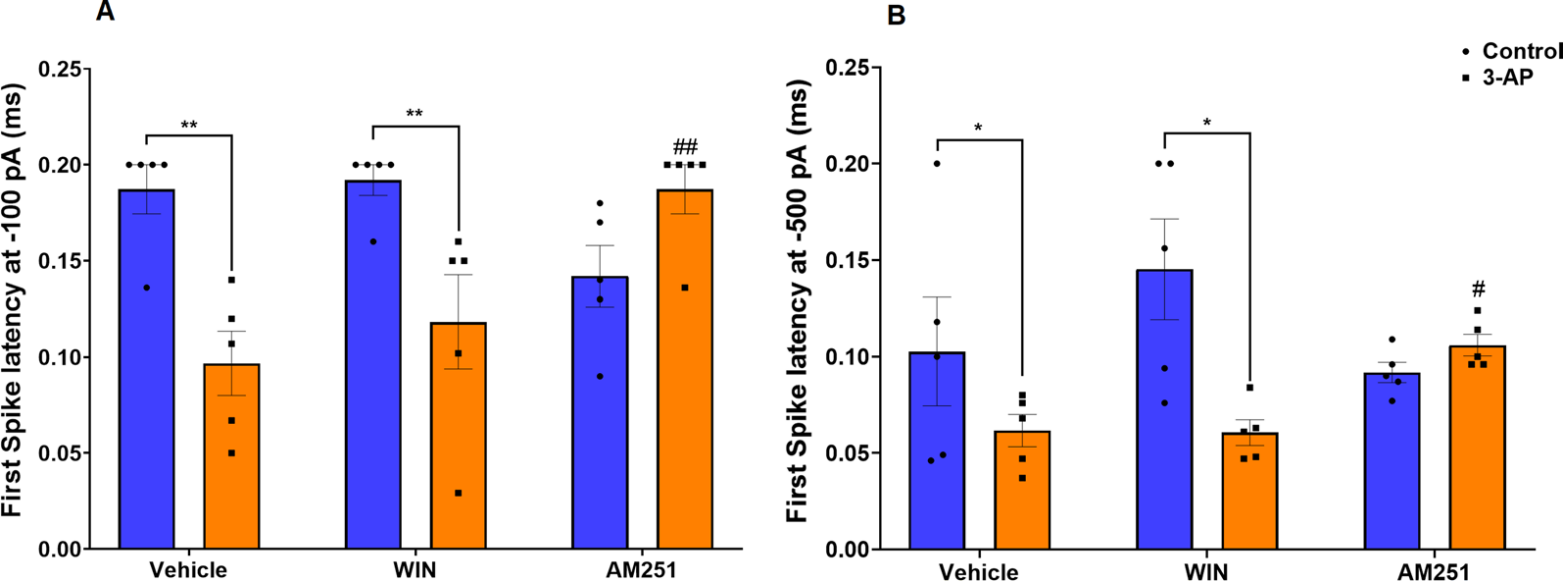
Antagonist/agonist of CB1R effects on 3-AP-exposed cells' first spike latency at **A **-100 pA and **B** -500 pA. n = 6 cells/group; one cell from each rat. The data are expressed as mean ± SEM. *(P < 0.05), and **(P < 0.01) represent the significant difference with the same group with Vehicle (DMSO). #(P < 0.05), ##(P < 0.01) represents the significant difference versus 3-AP

Rebound is defined as membrane depolarization occurring at the offset of a hyperpolarizing stimulus and is one of several intrinsic properties that may promote rhythmic electrical activity. Purkinje cells from 3-AP (P < 0.001) and 3-AP + WIN (P < 0.001) exposed brain slices displayed a significantly higher number of rebound action potentials (F (2, 24) = 8.932, P = 0.0013) appearing following presence of a hyperpolarizing conditioning pulse (Fig. 6A) and a reduction in the first spike latency (F (2, 24) = 10.71, P = 0.0005), (Fig. 7A) at -100 pA (P < 0.01) compared to averages for these same parameters in Purkinje cells from Co and WIN groups, respectively. In Purkinje cells in the 3-AP treatment group, which were treated with AM (P < 0.05), conversely, a lower number of rebound action potentials was noted (Fig. 6A, B) as well as an enhanced first spike latency (Fig. 7A, B) at both -100 pA (P < 0.01) and -500 pA (P < 0.05) compared to 3-AP Purkinje cells.

The rebound of action potentials was significantly higher in Purkinje cells from the 3-AP and 3-AP + WIN groups when compared to that seen in the Co and WIN groups at both -100 pA (P < 0.001) and -200 pA (P < 0.05). Furthermore, there was a significantly lower rebound in Purkinje cells in the 3-AP + AM group at -200 pA and -500 pA compared to 3-AP cells. Therefore, exposure to AM was capable of reducing the heightened excitability of Purkinje cells associated with 3-AP treatment.

Exposing Purkinje cells to 3-AP led to an increase in rebound spikes, which is a sign of heightened excitability, and treating them with AM decreased the rebound spikes. Thus, our data shows that evoked and spontaneous activities can be reversed by AM.

**Fig. 8:**
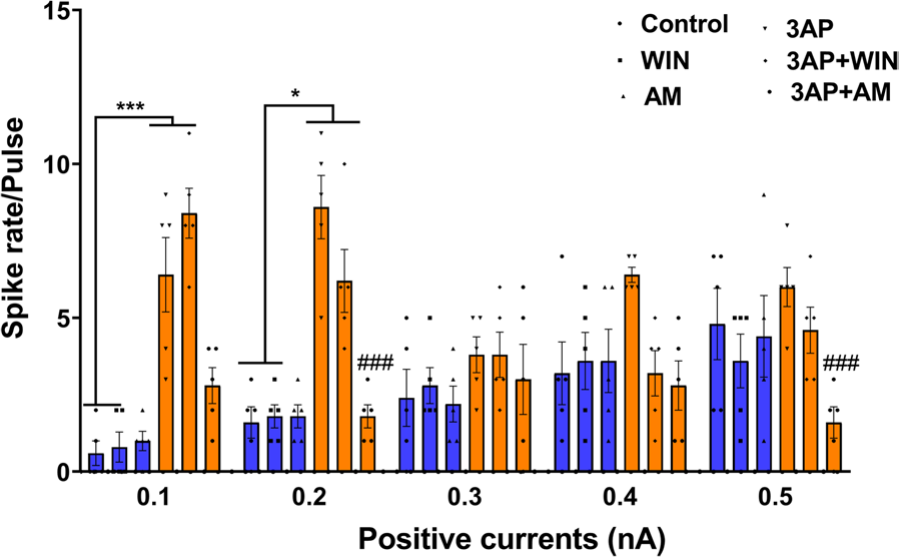
Population data representative of the effects on spike rate/pulse (0.1 nA–0.5 nA). n = 6 cells/group; one cell from each rat. The data are expressed as mean ± SEM. *(P < 0.05) and ***(P < 0.001) represent the significant differences with the same group with Vehicle (DMSO). ###(P < 0.001) represents the significant difference versus 3-AP

Furthermore, the prolonged, time-dependent changes evident in the spike rate/pulse of action potential after the increase in the current step from 0.1 nA to 0.5 nA indicate that an adaptation occurs in the action potential in all groups. Particularly, we did note a significant difference in the adaptation seen in the 3-AP groups (Fig. 8).

**Fig. 9:**
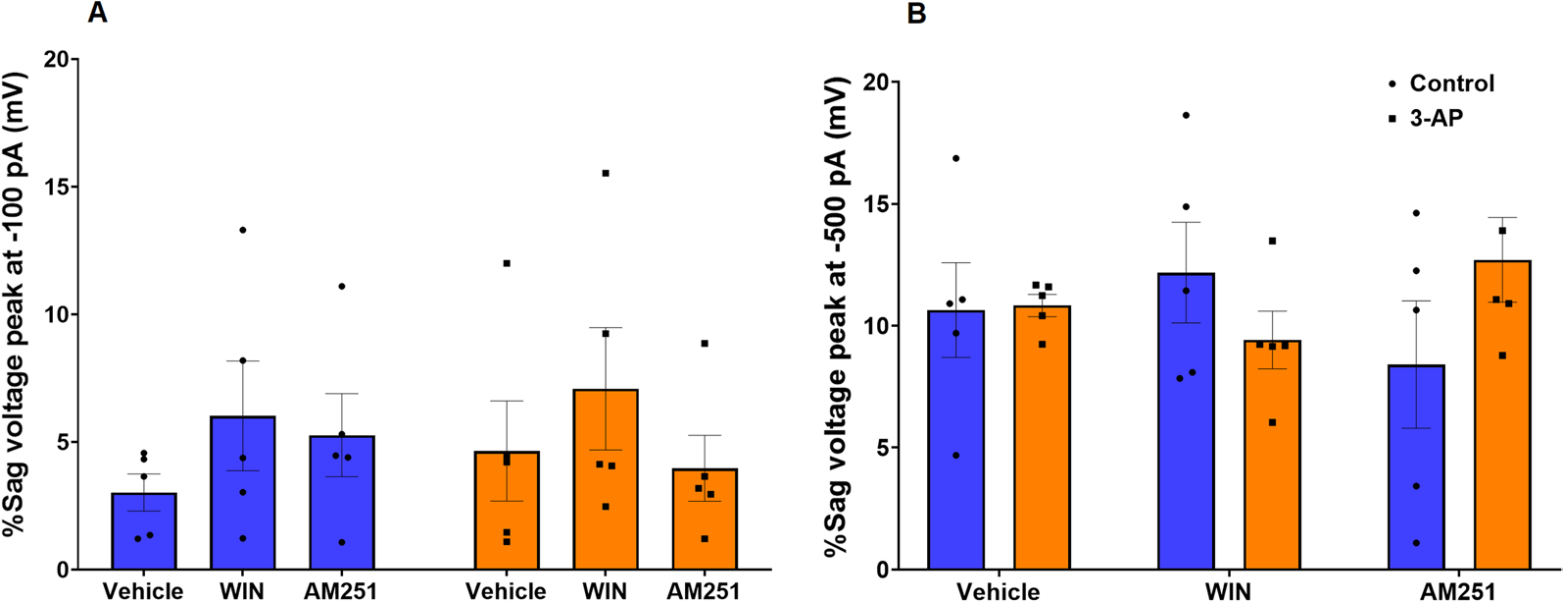
The percentage of the sag potential was determined at **A** -100pA and **B** -500pA. n = 6 cells/group; one cell from each rat. The data are expressed as mean ± SEM

The sag voltage was studied to determine the effects of CB1R modulation on Ih currents (hyperpolarization-activated currents) in Co and 3-AP treated slices. Sag is considered an indicator of the kinetics and amplitude of Ih relative to the membrane time constant. After the application of hyperpolarizing current pulses (-100 pA (F (2, 24) = 0.3777, P = 0.6894) and -500 pA (F (2, 24) = 1.931, P = 0.1668)) the %sag voltage showed no significant difference in any group (Fig. 9). This finding suggests that the effects of cannabinoids on ataxia might not involve actions at Ih.

## Discussion

The sequence of events that occur during 3-AP exposure that result in permanent damage to the Purkinje cells is poorly understood. Here, we utilized 3-AP-exposure, which is a valid model for inducing [7, 20] damage to rat Purkinje cells associated with behavioral signs of ataxia, in order to understand alterations in the electrophysiological properties of cerebellar Purkinje cells. 3-AP caused marked changes to the activity of Purkinje cells, consistent with previous studies showing that 3-AP can regulate neuronal excitability [4, 24]. In some aspects, the electrophysiological properties of 3-AP-exposed Purkinje cells were similar to those seen in controls, as we saw no differences in Rin, rebound (-500 pA), or %Sag voltage (-100 pA and -500 pA). However, there were differences, as Purkinje cells from the 3-AP group exhibited a higher firing frequency and greater AP amplitude. These electrophysiological changes were accompanied by an increase in putative excitatory spontaneous synaptic activity. While we did not identify the synaptic activity as glutamatergic, with the recording conditions we utilized synaptic events indicative of inward currents were unlikely to be GABA mediated, and the kinetic profiles of these events suggested involvement of an AMPA receptor. Further, the lower rheobase suggested heightened excitability in Purkinje cells exposed to 3-AP. Heightened excitability was also seen in 3-AP-exposed cells, as hyperpolarization was strongly and positively associated with increased spike probability. The effects of 3-AP on the frequency, AP amplitude, ISI, rebound spikes, and rheobase were reversed by the administration of AM251. Therefore, using 3-AP-exposed neurons in the current study, we found that Purkinje cells had a higher excitability, which was modulated by blocking CB1Rs.

WIN, a cannabinoid agonist, exacerbated the 3-AP-induced effects by further increasing the frequency and reducing the rheobase as well as increasing the EPSCs amplitudes exhibiting hyperexcitability. Previous studies have shown that CB1Rs are strongly expressed in the cerebellum and regulate Purkinje cell synaptic signaling [25, 26]. Accordingly, CB1Rs modulate GABA, glutamate, and other neurotransmitters throughout the cerebellum, thereby controlling movement and contributing to some movement disorders, such as ataxia [13–15]. From the reports above, our findings demonstrated a higher excitability in the CB1R agonist exposure that suggests alterations in glutamate/GABA might play a role in CB1R-associated hyperexcitability, and it might be possible to modulate these changes by blocking these receptors.

The amplitude of the AHP can regulate the ISI and thus determine the pace of spontaneously firing neurons. The larger the AHP or the greater the degree of hyperpolarization, the slower the cell will reach the firing threshold, which is reflected as a decrease in excitability, whereas if the AHP is smaller, the cell can reach the firing threshold more quickly, which indicates an increase in excitability. However, we observed an enhancement in AHP in 3-AP Purkinje cells with an increase in firing rate, which does not suggest such a direct relationship. We interpret our data to suggest that the greater amplitude AHP increases the availability of Na^+^ channels through more rapid removal of their inactive phase, which facilitates the generation of burst discharge and thus increases excitability [27].

AHP is a principal feedback mechanism for the control of the frequency and pattern of neuronal firing [28]. At high firing frequencies, AHP amplitudes and thresholds would be expected to decrease, but our results showed no decrease in AHP amplitudes. One potential explanation for our data is that the combination of the decrease in RMP (more depolarized Purkinje cells) with the increase in AHP in 3-AP-exposed rats prevented the threshold from changing. Furthermore, the decrease in ISI indicates that the kinetics of the Na^+^ channels have changed, leading to a more rapid opening and closing of these channels and contributing to the decrease in ISI. The AHP amplitude was significantly larger in neurons from 3-AP-exposed animals, but other parameters such as the Rin during the rising or decay phase of the action potential were not different between groups. Therefore, it seems likely that voltage-gated Na^+^ channels are key regulators of the alteration in AHP. The involvement of Na^+^ channels could suggest that the fast/medium AHP might be involved; however, our data argue against a role for the medium AHP. The slow component of the AHP is mediated by Ih currents [29], and as our analysis showed that there were no significant differences in %sag, this suggests not only that Ih currents might not contribute to effects of 3-AP exposure but also provides indirect evidence that the medium AHP is not involved in the dysfunction induced by 3-AP.

A possible mechanism could be alterations in the 3-AP model of fast AHP (fAHP), which is mediated by the BK channel. BK channels are activated at negative membrane potentials, and their large conductivities depend on high Ca^2+^ levels, which affect the local membrane excitability. AHP and the regulation of Ca^2+^ spikes occur in cerebellar Purkinje cells through activity of BK channels expressed in dendrites, soma, and myelinated axons [30]. However, BK channels should not be considered strictly excitatory or inhibitory, as they can be inhibited or activated by pharmacological or genetic inhibition. In other words, BK channels can amplify the firing of quiet neurons and reduce the firing of overly active ones [31, 32]. Furthermore, recent studies indicated that the BK channel could represent an outstanding therapeutic target for the management of ataxia [33], as BK channel mutations cause ataxia [34]. Overall, based on previous studies and our findings, it appears that BK channels are possible mediators of the effects of 3-AP on AHP and could be involved in mechanisms underlying ataxia. Therefore, future experiments should examine whether impairment of BK channels leads to changes in the excitability of ataxic Purkinje cells to determine the role played by these channels in ataxia.

The electrophysiological alterations described above were absent in Purkinje cells from rats that were treated with AM251. A potential mechanism might be the inhibition of Na^+^ channels by AM251, which reduces neuronal excitability through blockade of voltage-sensitive Na^+^ channels in the brain [35]. Another non mutually exclusive possibility is that effects could be due to (endo)-cannabinoid-mediated modulation of BK channels as it has been reported that (endo)-cannabinoids directly interact with and can modify BK channel activity [36]. As a result, AM251’s effects on modulating excitability changes seen in the 3-AP Purkinje cells could be via modulation of BK function.

In conclusion, dysfunction of Purkinje cells in the cerebellum contributes to the clinical signs and symptoms of ataxia. Based on our findings, we conclude that effects of 3-AP exposure alter functioning of Purkinje cells. One caveat of our study was that we were not able to determine whether dysfunctions of Purkinje cells associated with 3-AP were mediated through direct effects on the Purkinje cells or on inputs directed to these cells. However, as our intention was to determine whether the excitability of Purkinje cells was altered by 3-AP treatment, whether effects were direct or indirect awaits further experiments. Our data also suggest that an antagonist of CB1R could be effective in modulating the devastating cellular effects of 3-AP. Although the mechanism responsible for this protection is unknown, findings with CB1R antagonists in this study could be relevant for future therapies for ataxia.

## Data Availability

The datasets used or analyzed during the current study are available from the corresponding author on reasonable request.

## Code availability

All software applications used are included in this article.
